# A Novel Method Linking Antigen Presentation by Human Monocyte-Derived Macrophages to CD8^+^ T Cell Polyfunctionality

**DOI:** 10.3389/fimmu.2013.00389

**Published:** 2013-11-21

**Authors:** Kirsty R. Short, Emma J. Grant, Marloes Vissers, Patrick C. Reading, Dimitri A. Diavatopoulos, Katherine Kedzierska

**Affiliations:** ^1^Department of Microbiology and Immunology, University of Melbourne, Melbourne, VIC, Australia; ^2^Laboratory of Pediatric Infectious Diseases, Department of Pediatrics, Radboud University Medical Centre, Nijmegen, Netherlands; ^3^WHO Collaborating Centre for Reference and Research on Influenza, Parkville, VIC, Australia

**Keywords:** CD8^+^ T cells, antigen presentation, macrophages, influenza virus, polyfunctionality

## Abstract

To understand the interactions between innate and adaptive immunity, and specifically how virally infected macrophages impact T cell function, novel assays examining the ability of macrophages to present antigen to CD8^+^ T cells are needed. In the present study, we have developed a robust *in vitro* assay to measure how antigen presentation by human monocyte-derived macrophages (MDMs) affects the functional capacity of autologous CD8^+^ T cells. The assay is based on the polyfunctional characteristics of antigen-specific CD8^+^ T cells, and is thus called a Mac-CD8 Polyfunctionality Assay. Following purification of monocytes and their maturation to MDMs, MDMs were pulsed with an antigenic peptide to be presented to CD8^+^ T cells. Peptide-pulsed MDMs were then incubated with antigen-specific CD8^+^ T cells in order to assess the efficacy of antigen presentation to T cells. CD8^+^ T cell polyfunctionality was assessed by staining with mAbs to IFN-γ, TNF-α, and CD107a in a multi-color intracellular cytokine staining assay. To highlight the utility of the Mac-CD8 Polyfunctionality Assay, we assessed the effects of influenza infection on the ability of human macrophages to present antigen to CD8^+^ T cells. We found that influenza infection of human MDMs can alter the effector efficacy of MDMs to activate more CD8^+^ T cells with cytotoxic capacity. This has important implications for understanding how the virus-infected macrophages affect adaptive immunity at the site of infection.

## Introduction

A robust CD8^+^ T cell response represents an essential component of controlling viral infections of the respiratory tract, including infection with influenza A virus (IAV). Each year seasonal outbreaks of IAV cause approximately 500,000 deaths worldwide (1). However, if a novel IAV strain enters a human population, and there is no pre-existing immunity, a global pandemic can result. Such pandemics can potentially cause millions of IAV-associated fatalities, as was observed during the 1918–1919 Spanish influenza virus pandemic (2). Unlike an antibody response, an effective CD8^+^ T cell response can offer heterosubtypic immunity against IAV, protecting individuals against both the current circulating IAV strain and novel pandemic strains (3). Thus, developing techniques to assess CD8^+^ T cell activation in humans, in particular in the context of an IAV infection, represents an important component of understanding the host anti-viral immune response.

Influenza A virus primarily infects and replicates within epithelial cells of the respiratory tract. IAV can also infect dendritic cells (DCs) and macrophages, although such infections are typically considered to be abortive (4). IAV infection of human DCs affects the subsequent induction of CD8^+^ T cell responses (5). Similarly, IAV infection of macrophages may affect antigen presentation to CD8^+^ T cells at the site of infection. The ability of macrophages to present viral antigen to CD8^+^ T cells represents an essential component an effective anti-IAV immune response, as unlike epithelial cells, macrophages are able to induce both CD8^+^ T cell cytolysis and cytokine production (6). Consistent with a role for macrophages in the CD8^+^ T cell response, impaired macrophage function in mice is associated with a diminished CD8^+^ T cell response and increased susceptibility to IAV infection (7). Due to the longevity of macrophages (8), the ability of a viral infection to alter macrophage-CD8^+^ T cells interactions may have long-term consequences for the adaptive immune response of the host.

Currently, the majority of studies investigating macrophage-CD8^+^ T cell interactions have focused on murine models of infection (6, 7). This may reflect the fact that to the best of our knowledge, no assay has been developed to assess the ability of human macrophages to present antigen to CD8^+^ T cells. This is despite the fact that such assays have been established to examine human DC-CD8^+^ T cell interactions (5). Here, we present a simple and robust *in vitro* assay to assess CD8^+^ T cell polyfunctionality following antigen presentation by human monocyte-derived macrophages (MDMs). To demonstrate the utility of this assay, we used this method to assess the effects of IAV infection of human MDMs on CD8^+^ T cell polyfunctionality. This assay can be further utilized to understand the impact of specific viruses, bacteria, parasites, and tumors on human macrophage-CD8^+^ T cell interactions.

## Materials and Methods

### Virus strain

The GFP-labeled influenza virus strain A/PR8-GFP/8/34 (GFP-PR8/34; H1N1) (9) was used in all experiments. Virus stocks were prepared in embryonated eggs and titers of infectious virus were determined by three independent plaque assays on Madin-Darby Canine Kidney (MDCK) cells (10).

### Isolation of human peripheral blood mononuclear cells

Human peripheral blood mononuclear cells (PBMCs) were isolated from blood obtained from anonymized buffy coats of healthy donors who consented to the use of their blood for scientific research (Sanquin, The Netherlands or Australian Red Cross, Australia). Where relevant, experiments were approved by the Ethics Committee of the University of Melbourne, Australia. PMBCs were isolated from buffy coats by density gradient centrifugation using Lymphoprep (Axis-Shield, Norway). PBMCs were then washed, resuspended in 10% DMSO (v/v) with heat-inactivated FCS and frozen in liquid nitrogen. Alternatively, washed PBMCs were used fresh and monocytes were isolated by adherence as described below.

### Differentiation, infection, and analysis of human MDMs

Peripheral blood mononuclear cells were resuspended in RPMI-1640 supplemented with 2% heat-inactivated human sera (Sigma, USA) and 5 × 10^6^ cells (per well of a 24-well plate) or 5 × 10^7^ cells (per 25 cm^2^ tissue culture flask) were seeded. Monocytes were then allowed to adhere for a minimum of 1 h at 37°C. Wells/flasks were subsequently washed three times in PBS to remove non-adherent cells. Adherent monocytes were differentiated into MDMs by incubation for 6–7 days at 37°C in RPMI + 10% FCS (RF10). MDMs were subsequently washed and infected with IAV (MOI of 0.01) or mock (RPMI-1640) for 1 h at 37°C. Virus was then removed and cells were incubated for 16 h in RF10. MDMs were subsequently washed and stained with the relevant antibody (see Table 1), fixed and analyzed on a FACS Canto flow cytometer (BD Biosciences) with FACSDiva software (BD Biosciences). Collected samples were analyzed with FLOWJO, version 8.8.7 (TreeStar Inc.).

**Table 1:** **Antibodies used in the study**.

	Company	Fluorophore	Clone
CD14	BD Pharmingen	V500	MSE2
HLA-DR	BD Pharmingen	PE-Cy7	G46-6
IL18R	R&D	PE	132029
CD163	R&D	APC	215927
CD107a	eBioscience	FITC	eBioH4A3
CD3	eBioscience	PE-Cy7	UCHT1
CD8	BD Pharmingen	PerCP-Cy5.5	S1
IFN-γ	BD Pharmingen	PE	25723.11
TNF-α	BD Pharmingen	APC	6401.1111
HLA-A2	eBioscience	APC	BB7.2

### Cytokine production

Levels of cytokines produced by MDMs were measured using the relevant human ELISA Kits (Sanquin, The Netherlands) at 16-h post-IAV or mock infection. IFNα production was measured using a human IFNα ELISA Ready-SET-Go! kit (eBioscience, San Diego, CA, USA).

### Mac-CD8 polyfunctionality assay

**Figure 1:**
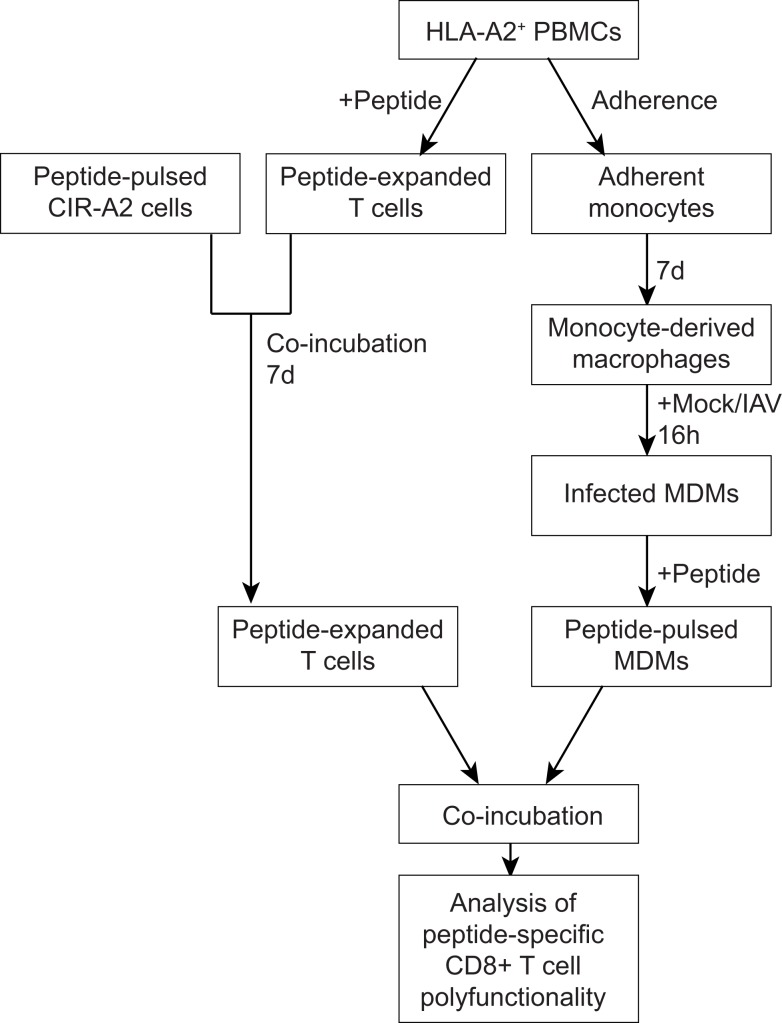
**An outline for the novel assay measuring interactions between human MDMs and CD8^+^ T cells**. PBMCs were isolated from the buffy coats of healthy donors and were screened for HLA-A2 expression. Monocytes were isolated by plastic adherence, whereas peripheral blood lymphocytes were used for T cell assays. Monocytes were then differentiated into macrophages, infected influenza virus (or mock-infected), and pulsed with peptide. Peripheral blood lymphocytes amplified *in vitro* following incubation with peptide-pulsed C1R-A2 cells. Peptide-pulsed MDMs were co-incubated with peptide-specific T cells and CD8^+^ T cell polyfunctionality was assessed by flow cytometry.

The methodology used to assess antigen presentation by human MDMs is shown in Figure 1. In these experiments, the Epstein–Barr virus (EBV) derived, HLA-A2 restricted, peptide EBV-BMLF1_280–288_(GLCTLVAML) (GLC) (11) was used. GLC was selected in place of an IAV peptide so that antigen presentation was independent of the IAV peptides processed and presented as a result of infection. In subsequent experiments, we also validated our new methodology using the IAV-derived, HLA-A2 restricted, peptide GIL M1_58–66_ GILGFVFTL.

#### Generation of peptide-specific CD8^+^ T cell lines

Firstly, HLA-A2^+^ human PBMCs were identified by staining of PBMCs with the HLA-A2^+^ BB7.2 mAb (a kind donation from the McCluskey Laboratory, University of Melbourne) and subsequent FACS analysis. To *in vitro* amplify CD8^+^ T cells specific to GLC or GIL, 1 × 10^7^ HLA-A2^+^ PBMCs were thawed in RF10 and washed twice in RPMI-1640. Approximately 3.3 × 10^6^ PBMCs were then incubated for 1 h with 10 μM GLC or GIL at 37°C. PBMCs were then washed twice in RPMI-1640 and added to the remaining PBMCs. Cells were subsequently incubated at 37°C and were supplemented with IL-2 twice weekly from day 3 (20 U/mL, Cetus, USA). Seven days later C1R cells that were transfected with HLA-A2 (C1R-A2) were washed twice in RPMI-1640 and incubated for 1 h with GLC or GIL (10 μM) at 37°C. C1R-A2 cells were then resuspended in RF10 and gamma irradiated at 8000 rad. Following irradiation, C1R-A2 cells were washed and added to T cell cultures at a ratio of 1:10 (C1R-A2 cells:T cells) in order to increase the number of EBV-specific T cells.

#### Assessment of peptide presentation by MDMs

Seven days later the ability of infected/stimulated MDMs (grown in a 24-well plate) to present GLC or GIL to autologous, EBV-specific CD8^+^ T cells was assessed by flow cytometry. MDMs were washed once with RPMI-1640 and incubated for 1 h with GLC or GIL (10 μM) at 37°C. Alternatively, MDMs were pulsed with 10 μM NS3_1073–1081_CINGVCWTV (CING), a HLA-A2 restricted peptide derived from Hepatitis C virus (HCV) (12) or 10 μL DMSO. MDMs were then washed and co-incubated with 6 × 10^5^ *in vitro* amplified EBV-specific T cells (per well) for 6 h at 37°C in the presence of anti-CD107a (Table 1), monensin (5 μM; Sigma) and BD GolgiPlug (BD Biosciences, USA) (13). T cells were subsequently removed, washed, and stained for CD3 and CD8 (Table 1). T cells were then washed, fixed, and permeabilized using the Cytofix/Cytoperm Fixation/Permeabilization kit (BD Biosciences) and stained with anti-IFN-γ and anti-TNF-α (Table 1). Cells were then analyzed on a FACSCantoII or Fortessa with FACSDiva software (BD Biosciences). Collected samples were analyzed with FLOWJO, version 8.8.7 (TreeStar Inc., USA).

## Results and Discussion

To gain insight into interactions between components of innate and adaptive immunity, and specifically how virus-infected macrophages impact the functionality of CD8^+^ T cell responses, novel assays examining the ability of macrophages to present antigen to CD8^+^ T cells are needed. In the present study, we developed a robust *in vitro* assay to assess how antigen presentation by human MDMs affects the functional capacity of autologous CD8^+^ T cells. The assay is based on polyfunctional characteristics of antigen-specific CD8^+^ T cells, and is thus called a Mac-CD8 Polyfunctionality Assay. Polyfunctionality of CD8^+^ T cells is defined by simultaneous production of multiple anti-viral cytokines (e.g., IFN-γ, TNF-α, and IL-2) (14), and the killing efficacy (assessed by CD107a; a measure of degranulation) of CD8^+^ T cells (15). Such polyfunctional CD8^+^ T cells producing an array of anti-viral cytokines are important for effective virus clearance (16, 17).

**Figure 2:**
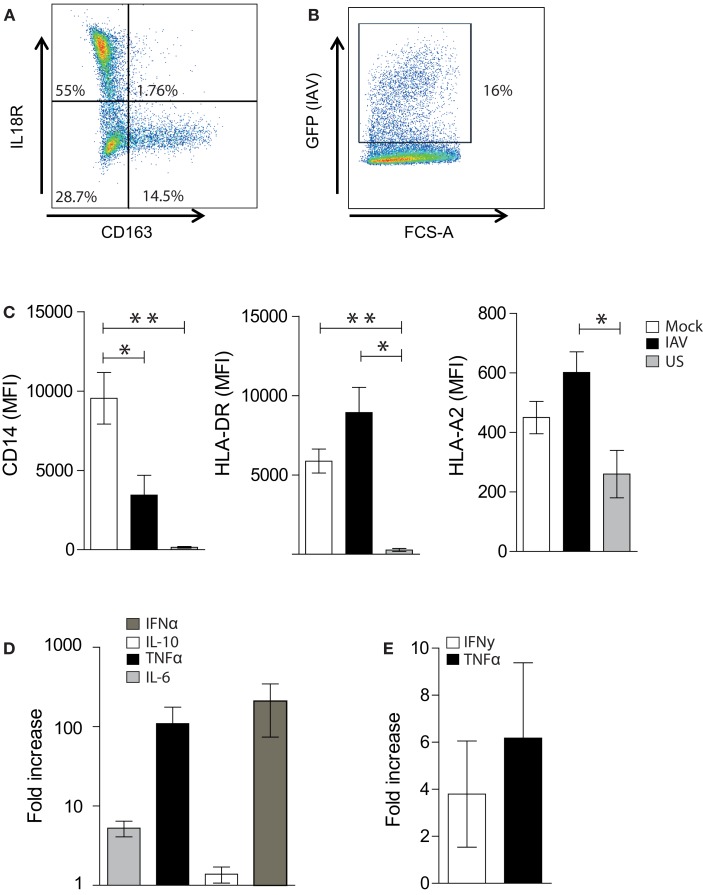
**Optimization of the Mac-CD8^+^ polyfunctionality assay**. **(A)** A representative FACS plot showing IL18R and CD163 expression of MDMs following incubation of adherent monocytes for 6–7 days at 37°C in RF10. Percentages represent the proportion of single-cells positive for the relevant antigen. **(B)** A representative FACS plot showing the rate of IAV infection 16-h post-infection at an MOI of 0.01. **(C)** Mean Fluorescent intensity (MFI) of CD14, HLA-DR, and HLA-A2 on single-cell MDMs 16 h post-IAV or mock infection. MFI values for unstained cells (US) are also shown. Data is pooled from a minimum of three independent donors. Statistical significance was assessed by a One-Way ANOVA with a Bonferroni post-correction and is denoted by one asterisk (*p* < 0.05) or two asterisks (*p* < 0.01). **(D)** Cytokine production 16 h post-IAV or mock infection of MDMs. Data are expressed as the fold increase in cytokines produced by IAV-infected MDMs relative to mock-infected MDMs. Data are pooled from three independent donors and are shown as the mean ± SEM. **(E)** Fold increase in the percentage of TNF-α^+^ or IFN-γ^+^ cells amongst CD3^+^ CD8^+^ cells following co-incubation with GLC-pulsed PBMCs, followed 1 week later by co-incubation with irradiated C1R-A2 cells pulsed with GLC (2× rounds of amplification). Data is expressed relative to the percentage of TNF-α^+^ or IFN-γ^+^ cells amongst CD3^+^ CD8^+^ cells following *in vitro* amplification by co-incubation with GLC-pulsed PBMCs (1× round of amplification). Data are pooled from two independent donors and are shown as the mean ± SEM.

**Figure 3:**
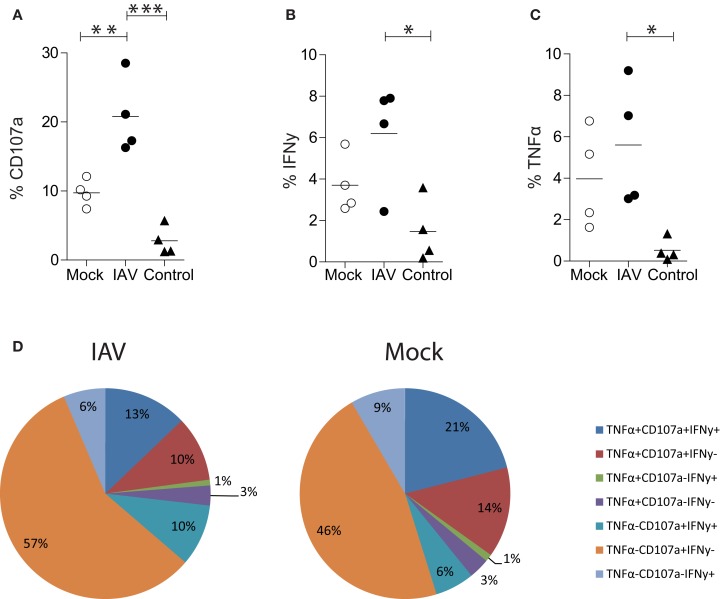
**Influenza A virus infection of human MDMs increases degranulation by EBV (GLC) specific CD8^+^ T cells**. **(A)** CD107a, **(B)** IFN-γ, or **(C)** TNF-α expression by single-cell, CD3^+^ CD8^+^ T cells following 6 h co-incubation with IAV- or mock-infected GLC-pulsed MDMs. Alternatively, T cells were incubated with MDMs pulsed with an irrelevant peptide (IP) derived from HCV (“control”). Data are pooled from four independent donors and statistical significance was assessed by a One-Way ANOVA with a Bonferroni post-correction and is denoted by one asterisk (*p* < 0.05), two asterisk (*p* < 0.01), or three asterisks (*p* < 0.001). **(D)** Polyfunctionality of CD8^+^ T cells following 6 h stimulation with peptide-pulsed MDMs. CD3^+^ CD8^+^ T cells expressing/producing combinations of CD107a, IFN-γ, and TNF-α from four independent donors are averaged and represented as a proportion of CD3^+^ CD8^+^ T cells.

In our study, we have used the polyfunctional characteristics of CD8^+^ T cells to assess the effect of IAV on the CD8-Mac interaction and the resulting CD8^+^ T cell responses (Figure 1). We isolated human peripheral blood monocytes from buffy packs, matured them to MDMs, infected with IAV or mock, stimulated with an antigenic peptide and assessed their antigen presentation to the responding CD8^+^ T cells by staining with mAbs to IFN-γ, TNF-α, and CD107a in a multi-color intracellular cytokine staining (ICS) assay (Figure 1). We have optimized both the antigen presentation by MDMs as well as culture conditions to ensure maximum expansion of antigen-specific CD8^+^ T cells. Subsequently, we utilized this novel Mac-CD8 Polyfunctionality Assay to understand the effects of IAV infection of human macrophages (Figure 2) on antigen presentation to CD8^+^ T cells. In these experiments, the EBV-derived peptide EBV-BMLF1_280–288_(GLCTLVAML) (GLC) was used for antigen presentation and T cell expansion to capture antigen presentation unrelated to influenza virus infection (Figure 3).

### Optimization of macrophage infection and T cell amplification

Macrophages represent a heterogenous population that can be broadly classified into pro-inflammatory “M1” macrophages and alternatively activated “M2” macrophages (although this definition typically represents a spectrum rather than two distinct groups) (18, 19). Thus, prior to assessing macrophage-CD8^+^ T cell interactions, we firstly characterized the M1 and M2 MDM populations induced by our *in vitro* culture conditions. It was evident that our methodology elicits predominately (at >50%) M1 macrophages (defined as IL18R^+^CD163^−^ cells) and a smaller set of M2 macrophages (<20%; defined as IL18R-CD163^+^) (Figure 2A) (19).

To assess the ability of virus-infected MDMs to present antigen to CD8^+^ T cells, we established an IAV infection model. We used an MOI of 0.01, which resulted in an average infection rate of approximately 15–20% (Figure 2B), as determined by GFP-positivity. This was associated with a significant decrease in CD14 expression but no alteration in MHCII and MHCI expression (Figure 2C). This decrease in CD14 expression is consistent with previous studies showing that PR8 decreases the expression of CD14 on human monocytes (20). Interestingly, influenza infection of MDMs resulted in a 100-fold and 10-fold increase in the production of TNF-α and IL-6, respectively, whilst no dramatic increase was observed in IL-10 production (Figure 2D). IFNα production was also increased approximately 100-fold following IAV infection. Thus, as a MOI of 0.01 altered macrophage function, we continued with this influenza virus infection regime to investigate macrophage-CD8 T cell interactions.

In order to increase the sensitivity of the assay and optimize the *in vitro* expansion of antigen-specific T cells, we introduced modifications to our standard *in vitro* culture and CD8^+^ T cell-mediated IFN-γ ICS assay (21). This was essential to assess CD8^+^ T cell polyfunctionality, as typically, only a subset of CD107a-positive CD8^+^ T cells produces cytokines (22). As shown in Figure 2E, the addition of a second round of *in vitro* amplification (in the presence of GLC-pulsed irradiated, C1R-A2 cells) resulted in an approximate fourfold increase in the number of IFN-γ^+^ CD8^+^ T cells detected following the presentation of GLC by MDMs. Similarly, the addition of a second round of *in vitro* amplification resulted in a sixfold increase in the number of TNF-α^+^ CD8^+^ T cells detected following presentation of GLC by MDMs (Figure 2E). As a result, T cells were cultured *in vitro* for 2 weeks prior to assessing macrophage-CD8^+^ T cell interactions.

### Antigen presentation to CD8^+^ T cells by human MDMs

Having optimized the conditions to assess antigen presentation by MDMs, we used the Mac-CD8 Polyfunctionality assay to determine how infection of MDMs with IAV affected antigen presentation. Specifically, we compared CD107a, IFN-γ, and TNF-α expression by CD8^+^ T cells following their incubation with IAV- or mock-infected MDMs presenting the GLC peptide. Our novel assay was sufficiently sensitive to detect significant increases in CD8^+^ T cell degranulation (CD107a expression) by IAV-infected MDMs relative to mock-infected MDMs (*p* < 0.01; Figure 3A). There was also a trend toward increased IFN-γ and TNF-α expression upon IAV infection, although these differences were not significant (*p* > 0.05; Figures 3B,C). There was limited CD8^+^ T cell degranulation or cytokine production observed following incubation of MDMs with an irrelevant HLA-A2^+^ restricted HCV peptide (CING), confirming the specificity of the Mac-CD8 Polyfunctionality assay (Figures 3A–C).

**Figure 4:**
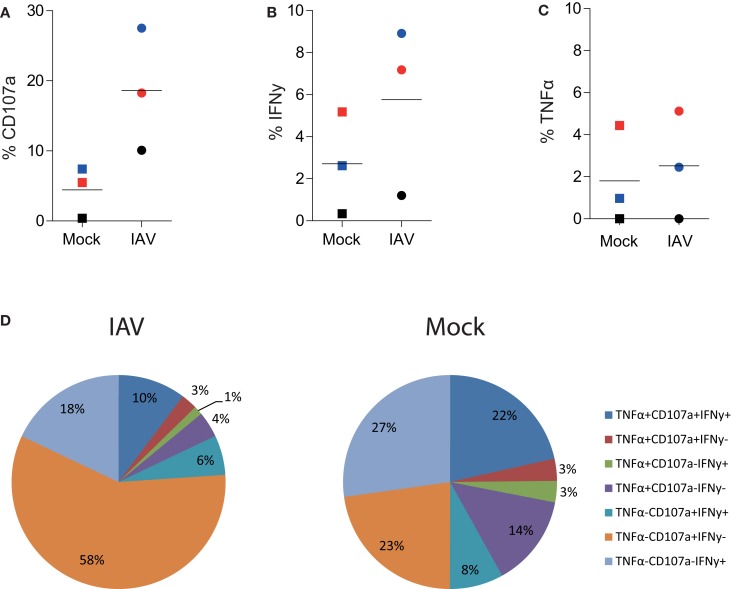
**Influenza A virus infection of human MDMs increases degranulation by influenza (GIL)-specific CD8^+^ T cells**. **(A)** CD107a, **(B)** IFN-γ, or **(C)** TNF-α expression by single-cell, CD3^+^ CD8^+^ T cells following 6 h co-incubation with IAV- or mock-infected M1-pulsed MDMs. The percentage of positive cells detected following co-incubation with mock-infected macrophages pulsed with 10 μL DMSO (instead of peptide) was subtracted from the relevant values. Data are pooled from three independent donors and each donor is indicated with a different color. **(D)** Polyfunctionality of CD8^+^ T cells following 6 h stimulation with peptide-pulsed MDMs. CD3^+^ CD8^+^ T cells expressing/producing combinations of CD107a, IFN-γ, and TNF-α from three independent donors are averaged and represented as a proportion of total CD3^+^ CD8^+^ T cells.

Importantly, this methodology provides data on the qualitative (in addition to quantitative) aspects of CD8^+^ T cells, termed polyfunctionality. Pooled results from four donors show that there was no difference in polyfunctionality observed between mock- and IAV-infected MDMs (Figure 3D). We observed preferential induction of CD8^+^ T cells with exclusive cytotoxic capacity (rather than cytokine-producers) by IAV-infected peptide-pulsed MDMs (Figure 3D). This hints that the main task of CD8^+^ T cells during influenza virus infection is to kill virally infected cells at the site of infection rather than produce anti-viral cytokines. To confirm that these findings were not restricted to the presentation of an EBV-derived peptide, we also performed the Mac-CD8 Polyfunctionality assay using an IAV-derived antigen (M1). Consistent with our previous data, we also saw a trend toward increased CD107a expression by CD8^+^ T cells following co-incubation with IAV-infected macrophages (relative to mock-infected macrophages) (Figure 4). Furthermore, we confirmed our findings of a preferential induction of CD8^+^ T cells with exclusive cytotoxic capacity by human MDMs following IAV infection (Figure 4). Interestingly, presentation of the M1 antigen by mock-infected MDMs resulted in a large number of T cells which produced IFN-γ in the absence of CD107a expression (Figure 4).

Taken together, we have demonstrated a novel *in vitro* assay to assess antigen presentation by human MDMs to autologous CD8^+^ T cells. This method shows that infection with IAV results in increased the presentation of both a model antigen and an IAV-derived antigen to CD8^+^ T cells. This assay could be adapted to assess how a variety of other viral infections may alter antigen presentation by human MDMs. It would also be possible to further modify this methodology to assess antigen presentation by human alveolar macrophages, in order to most accurately depict the cell-to-cell interactions that occur in the lung. Thus, in summary, the present manuscript provides a novel methodology that can be used to provide new insights to our understanding of the complex interactions that occur between the innate and adaptive immune response.

## Conflict of Interest Statement

The authors declare that the research was conducted in the absence of any commercial or financial relationships that could be construed as a potential conflict of interest.
